# Acute Severe Hyponatremia Following Use of “Detox Tea”

**DOI:** 10.7759/cureus.14184

**Published:** 2021-03-30

**Authors:** George Gillett, Natesh Shivakumar, Antonia James, Jonathan Salmon

**Affiliations:** 1 Adult Intensive Care Unit, John Radcliffe Hospital, Oxford University Hospitals National Health Service Foundation Trust, Oxford, GBR

**Keywords:** herbal supplements, neurology and critical care, intensive care, hyponatremia

## Abstract

A previously healthy 51-year-old woman presented to the ED with a one-week history of malaise, myalgia, unsteadiness, and an intermittent mild headache. Physical examination revealed neurological signs including a broad-based gait and bilateral low amplitude tremor. Laboratory testing of electrolytes revealed acute severe hyponatremia (115 mmol/L). Further, history-taking revealed increased urinary frequency following regular consumption of an over-the-counter detoxification tea product. The patient made a good recovery following admission to the intensive care unit. We identify similar cases in the literature and explore potential causal mechanisms. This case highlights the importance of enquiring about the use of supplementary health products when taking a history, and specifically identifies a growing number of reports of acute severe hyponatremia following the use of “detox teas”.

## Introduction

Alternative health products are prevalent in economically developed settings [1]. However, herbal products and dietary supplements are not regulated as pharmaceutical medicines and are often not disclosed to healthcare professionals [2-4]. Consequently, their effectiveness and adverse effects are understudied, despite plausibly having the potential to detrimentally affect health outcomes. Previous reports suggest that complementary and alternative medicine products may cause acute liver failure [5,6].

We report a case of a previously fit and well 51-year-old woman presenting with acute severe hyponatremia following consumption of an over-the-counter detoxification product. We identify similar cases in the literature and explore the potential mechanisms and significance of these cases. We believe such reports should prompt healthcare professionals to discuss complementary health products directly with patients and consider them when diagnosing unexplained symptoms.

## Case presentation

A 51-year-old female nurse presented to the ED following a one-week history of malaise, myalgia, unsteadiness, and an intermittent mild headache. Immediately prior to her presentation, the patient’s husband heard her fall and found her lying on the floor unresponsive, having vomited. This was accompanied by twitching of all four limbs, but no incontinence or tongue-biting. The episode lasted approximately five minutes. Following this, the patient became responsive to speech but seemed confused along with difficulty finding and articulating words. Her involuntary “twitching” motor symptoms continued until hospital presentation.

The patient’s past medical history was unremarkable. She reported no previous ill-health, took no regular prescribed medicines, denied recreational drug or alcohol use, and was a lifelong non-smoker. She had received a seasonal influenza vaccination ten days previously, indicated for her occupation as a nurse. The patient denied any recent fevers, respiratory or gastrointestinal symptoms. She had no recent travel history and had no personal contacts who had recently been unwell. 

On neurological examination, her Glasgow Coma Scale score fluctuated between 14 (confused) and 15, with an Abbreviated Mental Test score (AMTS) of 8. The patient followed three-stage commands and named bedside objects with ease, however her speech was staccato. Her pupils were unequal (Right > Left), with the right reacting sluggishly to light. The patient was noted to have a broad-based gait and bilateral low amplitude tremor. She exhibited bidirectional nystagmus, bilateral dysdiadochokinesis, bilateral posturing of the hands, and curling of the little fingers, although no drift was observed. She had generalized dystonia, which appeared more athetoid than choreic in nature, and titubation. Other aspects of the neurological examination were unremarkable. The patient’s tone and reflexes were symmetrical and power was good (MRC: 5/5) throughout the upper and lower limbs. Other systems examinations found no abnormalities. The patient was noted to be euvolemic, based on fluid status examination (capillary refill time, skin turgor, mucosae, peripheral temperature, and color), monitoring of hemodynamic vital signs, and biochemical tests (described below).

Clinical observations were SpO2: 94% on room air, respiratory rate: 24 breaths/min, temperature: 36.5 degrees, heart rate: 107 bpm, and blood pressure: 195/88 mmHg. Her heart rate and blood pressure settled on repeat readings (92 bpm and 114/81 mmHg, respectively). Urine output monitoring was only commenced on day 1, and was within the normal range (approximately 2 L/day).

Upon further history-taking, the patient reported starting an over-the-counter “detox tea” four weeks previously. She drank approximately two cups daily. During this time, she experienced increased urinary frequency. This frequency had increased progressively across the four-week period and in the days leading to admission. An online search of the product did not identify a precise ingredients list, although the product was described as containing White Tea, Puerh Tea, Oolong Tea, Green Tea, Rose Petal, Black Tea, Persimmon, Chamomile, Dandelion, and Ginger. Besides the “detox tea”, the patient denied any significant changes to her dietary intake.

Investigations

The patient’s admission blood gas showed serum lactate: 4.5 mmol/L, pH: 7.61, HCO3-: 43.2 mmol/L, glucose: 8.4 mmol/L, sodium: 111 mmol/L, potassium: 2.4 mmol/L. Laboratory testing of electrolytes confirmed low sodium: 115 mmol/L and potassium: 2.3 mmol/L. Additional results included urea: 9.2 mmol/L, creatinine: 85 umol/L, adjusted calcium: 2.64 mmol/L, magnesium: 0.6 mmol/L, bilirubin: 32 umol/L, alanine aminotransferase: 118 Int Unit/L, aspartate aminotransferase 708 Int Unit/L, alkaline phosphatase 87 Int Unit/L, albumin 35 g/L and thyroid-stimulating hormone: 0.77 munit/L. Her white cell count was 32.63 x 10^9^/L, platelets: 466 x 10^9^/L, and C-reactive protein: 8.7 mg/L. Coagulation tests revealed INR of 1.0, prothrombin time: 10.6 seconds, and activated partial thromboplastin time: 20.1 seconds. Paired serum and urine osmolality (taken 14 hours following admission and following intravenous fluid administration) were serum: 285 mOsm/kg and urine: 128 mOsm/kg. Urine sodium was <20 mmol/L.

The patient’s chest X-ray showed an opacity at the right lung base consistent with infection or aspiration. CT and MRI scans showed no intracranial pathology.

Treatment 

The patient was initially treated with intravenous ceftriaxone, acyclovir, and dexamethasone, which were all stopped after one dose. She was admitted to the intensive care unit for monitoring. Intravenous fluids (1L of 0.9% NaCl with 40 mmol KCl, followed by IL of 5% dextrose with oral potassium replacement) were administered for potassium correction and slow correction of sodium. This was achieved; repeat sodium was 126 mmol/L (day 1) and 130 mmol/L (day 2) and repeat potassium was 3.0 mmol/L (10 hours) and 3.1 mmol/L (16 hours). Magnesium was replaced orally and corrected to 0.87 mmol/L (day 1). Both her lactate and white cell count trended down throughout admission. Her repeat white cell count was 18.41 x 10^9^/L (day 1) and 15.65 x 10^9^/L (day 2), and repeat lactate was 1.5 mmol/L (eight hours) and 1.3 mmol/L (16 hours).

Clinically, the patient also improved across this period. Her neurological signs improved by day 1 and the patient was asymptomatic by day 2.

Outcome

Following clinical resolution and improvement in laboratory investigations, the patient was discharged home after a three-day admission. She was prescribed oral potassium replacement in the context of ongoing mild hypokalemia and advised to book a follow-up appointment with her general practitioner for further electrolyte monitoring. We discussed the diagnostic uncertainty around the case, the possible role of dietary supplements and advised against the continued use of the product in question. Following discharge, the patient continued to experience good health.

## Discussion

It was felt most likely that the patient’s neurological symptoms and probable seizure were precipitated by severe hyponatremia. The occurrence of a seizure prior to admission, although not witnessed by medical staff, fits with the described events and the patient’s elevated lactate and white cell count on admission.

The patient’s hyponatremia may have resulted from multiple different mechanisms. The most likely explanation appears to be that the “detox tea” product exhibited a diuretic effect, which may have led to hyponatremia and concurrent hypokalemia. Although this is difficult to elucidate without a comprehensive ingredients list, we note three recent cases in the literature of herbal supplementation products being associated with acute severe hyponatremia requiring admission to the intensive care unit [7-9]. Two previous cases specifically report that the herbal remedy taken contained valerian root, and speculate on its relevance to the etiology of the case [7,8]. While valerian root is not an advertised ingredient of the product in this case, the lack of a comprehensive ingredients list makes this a worthy consideration.

It is also possible that polydipsia contributed to the patient’s hyponatremia. In the three previous case reports, hyponatremia was deemed to be a consequence of excessive water intake [7-9]. This is a possible explanation in this case, although it is not entirely in keeping with the history described by the patient. The patient only reported drinking two extra cups of “detox tea” daily in the prior weeks, with no other changes to her dietary intake, which appears insufficient to elicit such a marked response.

Less likely mechanisms include malnutrition or corrected hypovolemic hyponatremia. It is possible that malnutrition led to the patient’s concurrent hyponatremia and hypokalemia, however this does not fit with the patient’s history. Furthermore, she did not appear clinically malnourished on admission and her albumin was within normal range, suggesting she had sufficient dietary intake. Alternatively, it is possible that since the paired serum and urinary results were taken 14 hours following admission, they represent the consequences of a correction of antidiuretic hormone (ADH) release following fluid administration. This explanation seems unlikely given that biochemical tests were not suggestive of hypovolemia and the patient appeared clinically euvolemic upon admission. Furthermore, this explanation would not explain the patient’s hypokalemia. Finally, laboratory testing indicated liver injury, which represents another potential cause of hyponatremia. Although this mechanism is unlikely in this case, it is a worthy consideration given that complementary and alternative medicine products have previously been associated with acute liver failure [5,6]. 

A recent review of detoxing diets including “detox teas” found that such regimes suffer from poor quality evidence and lacked randomized controlled trials investigating either efficacy or adverse outcomes [10]. To our knowledge, this is the fourth case report describing a possible association between “detox teas” and acute severe hyponatremia causing serious neurological symptoms. We suggest these cases should prompt caution among members of the public using such products and encourage healthcare professionals to enquire about the use of supplements when caring for patients with unexplained symptoms.

## Conclusions

In conclusion, we describe the case of a previously fit and well 51-year-old woman presenting with neurological symptoms secondary to acute severe hyponatremia in the context of recent use of a “detox tea” product. Plausible mechanisms include a potential diuretic effect of the “detox tea” and possible polydipsia. Given that four case reports have observed a potential relationship between “detox teas” and acute severe hyponatremia, healthcare professionals and members of the public should be mindful of their potential health consequences. Where possible, such products should be rigorously investigated before being endorsed by medical practitioners. Finally, healthcare professionals should specifically enquire about the use of supplementary products when taking a medical history, especially as patients may not volunteer such information.
